# Supplementary material for: Family Functioning as an Explanatory Factor of Empathic Behavior in Argentine Medical Students
Source: Behav Sci (Basel). 2023 Apr 24;13(5):356. doi: 10.3390/bs13050356 (PMC10263231; doi:10.3390/bs13050356)

Family Functioning as an Explanatory Factor of Empathic Behavior in Argentine Medical Students

María J. Ulloque, Silvina Villalba, Gabriela Foscarini, Susana Quinteros, Aracelis Calzadilla-Núñez, Alejandro Reyes-Reyes and Víctor Díaz-Narváez

Figure S1. Two-factor model (latent variable structure) of the FACES-15.

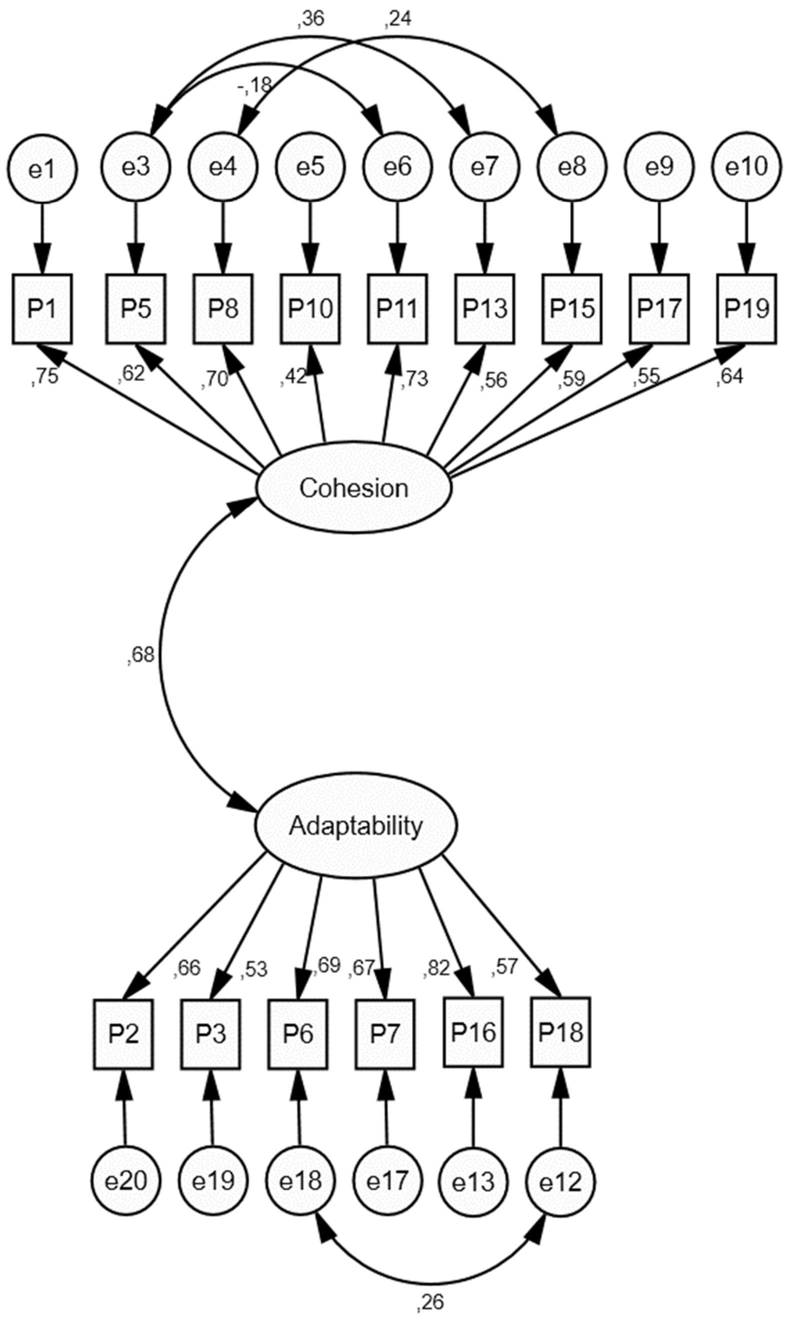

Supplement: Supplementary file 1 [file behavsci-13-00356-s001.zip › behavsci-2168753-supplementary.pdf]
